# The mediating role of nicotine dependence in the relationship between marital satisfaction and willingness to quit smoking: A cross-sectional study

**DOI:** 10.18332/tid/219364

**Published:** 2026-05-28

**Authors:** Yaoqi Zhang, Lingling Huo, Yani Wang, Yiqing Huang, Rui Wang, Nan Jiang, Yi Guo, Fei Qi, Shanpeng Li

**Affiliations:** 1 Department of Epidemiology and Health Statistics, School of Public Health, Qingdao University, Qingdao, China; 2 Qingdao West Coast New District Center for Disease Control and Prevention, Qingdao, China; 3 Qingdao Municipal Center for Disease Control and Prevention, Qingdao, China; 4 Peking University Clinical Research Institute, Peking University First Hospital, Beijing, China

**Keywords:** smoking cessation, marital satisfaction, nicotine dependence, mediating effect, structural equation modeling

## Abstract

**INTRODUCTION:**

Previous studies have established a link between marital relationship quality and smoking cessation behavior, but the mediating role of nicotine dependence has not been fully explored. The purpose of this study was to explore the association between marital satisfaction and willingness to quit smoking, and to test the mediating role of nicotine dependence in this association.

**METHODS:**

This study was conducted among students in grades 1 to 5 at 17 pilot elementary schools in Qingdao, China. It is a cross-sectional analysis study that collected data in July 2022. The sample consisted of families in which the father smoked and the mother did not. Researchers underwent standardized training before data collection. Parents were screened, and both spouses completed corresponding questionnaires, ultimately yielding 950 valid matched questionnaires. Structural equation modeling using AMOS was employed to test the mediating effect of nicotine dependence.

**RESULTS:**

Path analysis revealed that marital satisfaction was negatively associated with nicotine dependence (β= -0.091; 95% CI: -0.172 – -0.014). Willingness to quit smoking was positively related to marital satisfaction (β=0.063; 95% CI: 0.001–0.129) and negatively associated with nicotine dependence (β= -0.394; 95% CI: -0.469 – -0.314). Moreover, the indirect effect of willingness to quit smoking on marital satisfaction via nicotine dependence was positive and statistically significant (β=0.036; 95% CI: 0.006–0.072). Bootstrap mediation tests revealed statistically significant direct and mediating effects, with the mediating effect accounting for 36.36% of the variance.

**CONCLUSIONS:**

The results supported that nicotine dependence had a mediating effect between marital satisfaction and willingness to quit smoking.

## INTRODUCTION

Smoking is significantly associated with the risk of multiple diseases, and quitting smoking helps reduce the risk of illness and death^1^. The hazards of secondhand smoke (SHS) affect multiple aspects of health, including the respiratory system, cardiovascular system, and long-term health risks^2^. Children are especially susceptible to the harms of secondhand smoke exposure^2^ which among minors remains widespread^3^. Creating smoke-free environments in households with minors is urgently needed. Cessation motivation is a strong independent predictor of quit attempts^4^, and smokers who are motivated to quit are more likely to achieve and sustain abstinence compared to those lacking such motivation^5^.

The family serves as a crucial setting for smoking cessation, with the marital relationship functioning as its core subsystem^6^. Understanding how marital relationships – particularly their central indicator, relationship satisfaction^7^ – influence this challenging health behavior change process has significant theoretical and practical implications and plays a vital role in helping smoking husbands quit.

A common finding is that high-quality intimate relationships are typically associated with lower rates of substance abuse and better health outcomes^8^. Marital conflict is linked to an increased risk of nicotine dependence^9^, while improved relationship satisfaction is associated with reduced levels of nicotine dependence^10^ and is correlated with smoking patterns^10^.

High marital satisfaction is typically accompanied by more positive supportive behaviors, such as emotional support and encouragement to quit smoking, while reducing negative controlling behaviors like criticism or coercion. Smokers exhibit a significantly lower probability of smoking in daily life^11^. Furthermore, it has been confirmed that on days with increased supportive behaviors, smokers’ smoking probability decreases^12^, and both partners report higher relationship satisfaction. This reinforces the immediate inhibitory effect of a high-satisfaction environment on smoking behavior^13^.

Nicotine dependence is a significant physiological and psychological factor influencing the willingness to quit smoking^14^. Individuals with high dependence experience higher relapse rates due to physiological and psychological addiction mechanisms such as withdrawal symptoms and cravings^14,15^. The greater the nicotine dependence, the lower the willingness to quit and the more difficult it becomes to quit^14^. Extensive research indeed supports this view, indicating that high nicotine dependence is significantly associated with lower quit motivation and success rates^15^.

Currently, there is a lack of internal theoretical mechanisms explaining how marital satisfaction influences the smoking cessation motivation of husbands. The pathway through which marital satisfaction affects smoking cessation motivation by influencing nicotine dependence levels has not been fully validated. Therefore, this study aims to examine the mediating role of nicotine dependence between marital satisfaction and smoking cessation motivation among husbands who smoke.

## METHODS

### Study design

This study was conducted in 17 pilot primary schools, grades 1 to 5, in Qingdao, involving smoking fathers and non-smoking mothers. Since the experiment was conducted after the sixth-grade students graduated, their parents could not be contacted through the school. Therefore, only the students from the first grade to the fifth grade were included in the study. The investigators conducted a preliminary screening of potential participants who met the inclusion criteria, and then conducted telephone and face-to-face interviews to obtain further information about smoking fathers. Fathers who met the inclusion criteria were asked whether they were willing to participate in the study. Those who refused to participate were excluded. The final list of respondents was formed by class. It is a cross-sectional analysis study that collected data in July 2022.

### Population and sample

The study was conducted in accordance with the ethical standards outlined in the Helsinki Declaration (1983). This investigation was approved by the Medical Ethics Committee of the Qingdao Center for Disease Control and Prevention, project number 2021-ZXJK-32. All measurements in this study were taken with the informed consent of the respondents’ parents/guardians. The researchers provided the participants with detailed information about the purpose, content, mode, benefits, and the lack of risk in participation, as well as the respondents’ rights and interests. After fully understanding the relevant content, they were invited to complete an online questionnaire. The study sample comprised student households where the father smoked, and the mother did not. Prior to the survey, investigators underwent standardized training and contacted parents via telephone or in-person interviews to conduct preliminary screening against inclusion and exclusion criteria and verify eligibility for the study.

Inclusion criteria were: 1) Both parents had lived with their child during the past one month; 2) The father smoked one or more cigarettes daily during the past one month, while the mother did not smoke; and 3) Proficiency with mobile phone functions. Exclusion criteria were: 1) severe heart, brain, lung, or blood disorders; 2) history of mental illness or other medical conditions preventing questionnaire completion; 3) father’s prior participation in smoking cessation programs before the study; and 4) divorced parents.

### Research instrument

Questionnaires were administered to both the wife and her smoking husband.

The husband’s version included:

1. Personal information: age, occupation, education level, and presence of chronic diseases. These factors served as control variables in the study to eliminate confounding effects. Age was divided into three groups: 25–34, 35–44, and ≥45 years. Education level was categorized into two groups: high school and lower, and college and higher. Occupation was classified into three categories: white-collar, blue-collar, and other. Chronic disease status was grouped into two categories: no disease and illness. White-collar workers were defined as those employed in professional, managerial, or administrative occupations; blue-collar workers were defined as those employed in manual labor, manufacturing, or construction trades.
2. The ‘Willingness to quit smoking’ variable was assessed using the ‘Willingness to quit’ dimension from the MTM scale, developed by Sharma – founder of the Multi-Theoretical Model of Health Behavior Change – specifically for smokers^16^. The scale has good reliability and validity^16^. Two questions measured cessation initiation intent: ‘How likely are you to quit smoking in the next few weeks?’; and maintenance intent: ‘How likely are you to go a week without smoking starting now?’. Responses used a five-point Likert scale (1–5), yielding a combined score range of 2–10 for cessation intent, where higher scores indicated stronger motivation. In this study, the Cronbach’s alpha value of the scale was 0.978.
3. Nicotine dependence was assessed using the Fagerström test for nicotine dependence^17^, this questionnaire is the most commonly used one to measure nicotine dependence, and its Chinese version possesses sufficient reliability and validity^18^. The scale contains 6 items: ‘How long do you smoke the first cigarette after waking up in the morning?’, ‘Are you having difficulty controlling the demand for smoking in many non-smoking places?’, ‘Which cigarette do you think you are most reluctant to give up?’, ‘How many cigarettes do you smoke every day?’, ‘Do you smoke more in the first hour after waking up in the morning than at other times?’, and ‘Do you still smoke when you are ill in bed?’. The total score range is 0–10. The higher the score, the stronger the nicotine dependence. In this study, the Cronbach’s alpha value of the scale was 0.730.

The wife’s version questionnaire includes:

1. Marital satisfaction, assessed using the five satisfaction subscales of the Investment Model Scale^19^. The Chinese version of the questionnaire exhibits good reliability and validity^20^. There are five items: ‘I am satisfied with our relationship’, ‘I have a better intimate relationship than others’, ‘I have an ideal intimate relationship’, ‘Our relationship makes me feel happy’, and ‘Our relationship well meets my needs for intimacy, companionship, etc.’. Responses are rated on a nine-point Likert scale ranging from 0 (strongly disagree) to 8 (strongly agree), with higher scores indicating greater satisfaction. In this study, the Cronbach’s alpha value of the scale was 0.939.

### Statistical analysis

Descriptive statistics for key variables were conducted using SPSS 27.0, with results presented as means with standard deviations (SD), and frequencies and percentages. Comparisons between two groups employed independent samples t-tests, while comparisons among multiple groups utilized one-way analysis of variance (ANOVA). Spearman’s correlation analysis was employed to assess variable relationships. To analyze the mediating role of nicotine dependence, Structural Equation Modeling (SEM) was performed with AMOS 27.0. Analyses were further adjusted for age, education level, occupation, and any chronic disease factors previously reported to correlate with willingness to quit smoking^21^. Model fit was assessed using the minimum difference in relative χ^2^ per degree of freedom (CMIN/DF), root mean square error of approximation (RMSEA), comparative fit index (CFI), good fit index (GFI), adjusted good fit index (AGFI), and Tucker-Lewis Index (TLI). Confidence intervals (CIs) were calculated for direct, indirect, and total effects. Participants (4.1%) with missing data were excluded from the analysis. All p-values were two-tailed, with p<0.05 considered statistically significant.

## RESULTS

### Descriptive analysis results

The demographic characteristics of the study participants are summarized in Table 1. Among the 950 respondents, the largest proportion of student fathers (67.7%) were aged 35–44 years. Education at the college level or higher accounted for 62.3% of the total sample. The majority of student fathers were blue-collar workers (45.7%). Among the surveyed fathers, 7.2% of the total sample had chronic illnesses. Significant differences were observed in marital satisfaction (p=0.039), nicotine dependence (p<0.001), and willingness to quit smoking (p=0.003) across education levels. Fathers in different occupations also showed significant differences in marital satisfaction (p=0.020).

**Table 1:** Smoking husbands’ demographic characteristics, cross-sectional study, Qingdao, China, July 2022 (N=950)

*Variables*	*n (%)*	*Marital* *satisfaction* *Mean (SD)*	*p*	*Nicotine* *dependence* *Mean (SD)*	*p*	*Willingness* *to quit* *smoking* *Mean (SD)*	*p*
**Age** (years)			0.133		0.598		0.319
25–34	155 (16.3)	34.39 (8.31)		2.03 (2.19)		6.50 (2.94)	
35–44	643 (67.7)	34.81 (7.72)		2.00 (2.20)		6.90 (2.99)	
≥45	152 (16.0)	33.34 (9.57)		2.20 (2.21)		6.90 (2.99)	
**Education level**			0.039		<0.001		0.003
High school and lower	358 (37.7)	33.78 (8.94)		2.36 (2.25)		6.47 (2.98)	
College and higher	592 (62.3)	34.95 (7.61)		1.84 (2.15)		7.06 (2.97)	
Occupation*			0.020		0.907		0.586
White-collar	287 (30.2)	35.10 (7.23)		2.01 (2.23)		6.98 (2.88)	
Blue-collar	434 (45.7)	34.79 (7.77)		2.03 (2.17)		6.81 (3.01)	
Other	229 (24.1)	33.22 (9.70)		2.09 (2.23)		6.71 (3.06)	
**Chronic diseases**			0.144		0.269		0.506
No disease	882 (92.8)	34.62 (8.06)		2.02 (2.19)		6.85 (3.00)	
Illness	68 (7.2)	33.12 (9.23)		2.32 (2.35)		6.60 (2.82)	

Independent samples t-tests and one-way ANOVA were conducted to detect group differences for categorical variables; statistical significance threshold p<0.05.

* White-collar were defined as those employed in professional, managerial, or administrative occupations; Blue-collar were defined as those employed in manual labor, manufacturing, or construction trades.

### Variable correlation analysis

We conducted Spearman’s rank correlation analyses on the total scores of each key variable (Table 2). The results revealed significant pairwise correlations among marital satisfaction, nicotine dependence, and willingness to quit smoking. Specifically, marital satisfaction was negatively correlated with nicotine dependence (r= -0.112, p<0.001) and positively correlated with willingness to quit smoking (r=0.098, p=0.003). Additionally, nicotine dependence was negatively correlated with willingness to quit smoking (r= -0.315, p<0.001).

**Table 2:** Descriptive statistics and correlational analysis of marital satisfaction, nicotine dependence, and willingness to quit smoking, cross-sectional study, Qingdao, China, July 2022 (N=950)

*Variables*	*Marital* *satisfaction*	*Nicotine* *dependence*	*Willingness* *to quit* *Smoking*	*Mean*	*SD*	*Min*	*Max*
Marital satisfaction	1.000			34.510	8.149	0	40
Nicotine dependence	-0.112***	1.000		2.040	2.201	0	10
Willingness to quit smoking	0.098**	-0.315***	1.000	6.830	2.982	2	10

Correlation analysis was conducted to assess the relationships between variables. *p<0.05,

** p<0.01,

*** p<0.001

### Mediation analysis

After controlling for age, education level, occupation, and presence of chronic diseases, the hypothesized model was tested using SEM. Results indicated χ^2^/ df=1.938, GFI=0.986, AGFI=0.974, TLI=0.948, CFI=0.968, and RMSEA=0.031, meeting the criteria for good model fit. Path analysis revealed that marital satisfaction was negatively associated with nicotine dependence (β= -0.091; 95% CI: -0.172 – -0.014). In contrast, willingness to quit smoking was positively related to marital satisfaction (β=0.063, 95% CI: 0.001–0.129) and negatively associated with nicotine dependence (β= -0.394; 95% CI: -0.469 – -0.314). Moreover, the indirect effect of willingness to quit smoking on marital satisfaction via nicotine dependence was positive and statistically significant (β=0.036; 95% CI: 0.006–0.072). Bootstrap mediation tests, as shown in Table 3, revealed statistically significant direct and mediating effects, with the mediating effect accounting for 36.36% of variance.

**Table 3:** Summary of the role of nicotine dependence as a mediator between marital satisfaction and willingness to quit smoking of mediation model effects, cross-sectional study, Qingdao, China, July 2022 (N=950)

*Effect*	*Variables*	*Marital satisfaction*		*Nicotine dependence*	
		*β*	*95% CI*	*β*	*95% CI*
Total effect	Nicotine dependence	-0.091	-0.172 – -0.014		
	Willingness to quit smoking	0.099	0.034–0.167	-0.394	-0.469 – -0.314
Direct effect	Nicotine dependence	-0.091	-0.172 – -0.014		
	Willingness to quit smoking	0.063	0.001–0.129	-0.394	-0.469 – -0.314
Indirect effect	Willingness to quit smoking	0.036	0.006–0.072		

β: standardized regression coefficient. In the mediation analysis, we further adjusted for other covariates, including age, education level, occupation, and any chronic disease.

**Figure 1:**
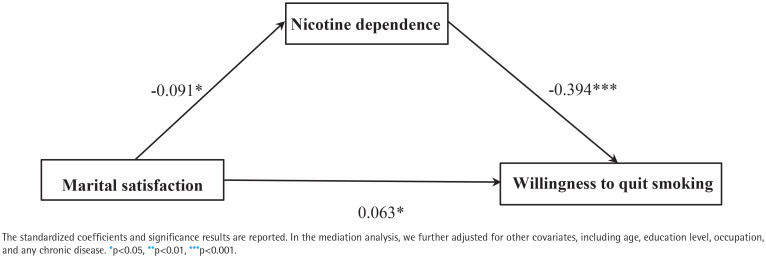
The mediating effect of nicotine dependence on marital satisfaction and husbands’ willingness to quit smoking was examined, cross-sectional study, Qingdao, China, July 2022 (N=950)

As shown in Figure 1, marital satisfaction showed a negative association with nicotine dependence (p<0.05) and a positive association with willingness to quit smoking (p<0.05). Nicotine dependence showed a negative association with willingness to quit smoking (p<0.001). After incorporating mediation into the model, the correlation between relationship satisfaction and cessation intention remained statistically significant, albeit with a slight decrease in strength, indicating the presence of partial mediation (β=0.063; 95% CI: 0.001– 0.129). Thus, the total effect can be decomposed into two components: the direct effect of marital satisfaction on willingness to quit smoking and the indirect effect mediated by nicotine dependence. The analyzed effects are detailed in Table 3.

## DISCUSSION

In this large cross-sectional study, we found that nicotine dependence had a significant mediating effect between marital satisfaction and the husband’s willingness to quit smoking. It shows that a considerable part of the impact of marital satisfaction on willingness to quit smoking is achieved by reducing nicotine dependence. The willingness to quit smoking, in addition to directly enhancing the willingness to quit smoking, can also reduce nicotine dependence by improving marital satisfaction, thus indirectly promoting the willingness to quit smoking.

Given the complex nature of family-based smoking cessation, relationship satisfaction is a crucial attribute that not only reduces smokers’ nicotine dependence but also enhances their willingness to quit^13^. Similar studies have confirmed that relationship satisfaction correlates with reduced use of addictive substances and is associated with smoking cessation behaviors^22^. However, other research conducted among general couples or mixed samples with and without children suggests that marital satisfaction may foster forgiveness, leading to greater tolerance of a partner’s smoking behavior^23^. The inconsistent findings may be primarily attributed to differences in study samples and family structures. The present study focused exclusively on couples with children, whereas previous studies often included a mixed sample of childless couples, romantic partners, and families with children, which weakened the family-driven effect on smoking cessation. In families with children, husbands may experience stronger health responsibilities toward their children and greater concerns about secondhand smoke exposure. Higher marital satisfaction may strengthen their willingness to maintain family health and harmony, thereby promoting willingness to quit.

In the family-based smoking cessation intervention, the quality of the marital relationship not only provides the key social emotional support for the smoking cessation process but also systematically affects the individual’s perception and evaluation of a variety of health behaviors. Specifically, positive marital interactions such as effective communication and emotional resonance can enhance smokers’ recognition of the benefits of behavior change and improve their overall health while reducing their risk perception of the difficulty of quitting smoking^24^. This reshaped perception will further strengthen and implement the specific health promotion behavior through the cooperative participation of spouses in the adjustment of common lifestyle^24^. This means that when smokers experience higher marital well-being and substantive support in the process of quitting smoking, they are more likely to internalize the behavior of quitting smoking into a positive choice that is responsible for their family and themselves, rather than just under external pressure^25^. This positive feedback from close relationships and shared health values can effectively promote the formation of stable and lasting internal change motivation^22^. Therefore, when designing and implementing family-based smoking cessation strategies, paying attention to and improving the quality of marital relationships and promoting constructive interaction between husband and wife, may be an important way to enhance the effect of the intervention by enhancing intrinsic motivation^11^.

Couples with high-quality marital relationships exhibit greater coordination and mutual support, leading to more desirable behaviors such as reduced smoking^26^. A highly satisfied partner living in a stable, positive, and emotionally supportive environment gains valuable psychological resources to cope with high-stress events like quitting smoking^27^. A highly satisfying marital relationship typically implies greater cohesion, smoother communication, and stronger stress coping abilities^27^. Within this low-conflict, high-support environment, smokers may rely less on smoking as a means to cope with stress or regulate emotions^25^. This reduced reliance may decrease smoking frequency and intensity at the behavioral level, potentially influencing physiological dependence levels^28^.

This study identified a potential relationship between marital satisfaction and husbands’ willingness to quit, partially mediated by nicotine dependence. Nicotine dependence drives persistent smoking behavior^29^, with higher dependence levels associated with lower quit success rates^30^. Through neurobiological mechanisms, nicotine dependence reinforces smoking behavior, significantly elevates health risks and comorbidity rates, and increases quit difficulty^31^. High nicotine dependence not only signifies stronger physiological dependence and withdrawal symptoms but also significantly diminishes smokers’ confidence in their ability to quit, thereby reducing their cessation motivation^32^. Nicotine dependence is not merely a physiological phenomenon but a deeply ingrained psychological state. High nicotine dependence correlates with poorer craving regulation and predicts higher relapse risk^33^. This finding aligns with prior research indicating that nicotine dependence is a key factor in motivating cessation intent, initiating cessation attempts, and achieving successful quitting^4^. Reducing nicotine dependence enhances cessation motivation, making it crucial for maintaining positive relationship quality in family environments aimed at reducing substance use^34^.

Although each family experiences varying levels of marital satisfaction, research indicates that marital satisfaction is a dynamic trait that can be cultivated and strengthened over time^35^. This underscores the importance of ongoing research to identify factors that enhance marital satisfaction, which can subsequently contribute to increasing smokers’ willingness to quit and their initiation of smoking cessation. Implementing community-based services such as counseling and relationship education can improve marital satisfaction^35^. Enhanced marital satisfaction cultivates and elevates smokers’ cessation motivation by reducing nicotine dependence through strengthened spousal support for quitting^11^.

These findings highlight the potential value of family-based smoking cessation programs that include components such as counseling or training aimed at enhancing marital satisfaction, which may, in turn, support willingness to quit smoking. Importantly, the results also point to factors beyond marital relationship quality: nicotine dependence plays a mediating role in the association between marital satisfaction and willingness to quit smoking. These findings suggest that marital satisfaction is related to willingness to quit smoking both directly and indirectly through its association with lower levels of nicotine dependence. Future research should consider employing longitudinal designs to further validate these findings. Additionally, future research should further investigate how varying degrees of nicotine dependence affect the role of marital satisfaction in shaping willingness to quit smoking, as understanding these dynamics could inform intervention strategies tailored to families with differing marital satisfaction levels.

### Limitations

The results and conclusions of this study must be considered within the context of its limitations. First, the use of a cross-sectional design precludes definitive conclusions regarding the direction of causality between variables. Second, this study is only conducted in pilot primary schools in Qingdao. It should be pointed out that the sample of this study is entirely composed of married couples. Therefore, the results may not directly apply to unmarried intimate partners or other intimate relationships. It should also be noted that over 62% of the smoking fathers in this study had a college degree or higher, indicating a predominantly well-educated sample. This imbalance may limit the generalizability of our findings to populations with lower levels of education. Future studies with more diverse educational backgrounds are warranted. The questionnaire was self-reported, and responses are thus subject to social desirability biases and recall bias. Fourth, despite adjusting for multiple covariates, including age, education level, occupation, and chronic disease status, residual confounding may persist due to unmeasured factors. Depressive symptoms were not assessed in this study, despite consistent evidence linking them to lower smoking cessation rates and reduced willingness to quit smoking36. Future studies should include these variables to better isolate the independent effects of the primary predictors. Finally, the mechanism for handling missing data is not completely missing at random (MCAR), which may lead to selection bias.

## CONCLUSIONS

This study found that nicotine dependence was associated with both marital satisfaction and willingness to quit smoking. Furthermore, due to the cross-sectional design, the direction of the observed associations cannot be determined. By linking individual physiological dependence to interpersonal dynamics, it deepens our understanding of health behavior change mechanisms. By repairing and strengthening intimate relationships, we may help cultivate supportive conditions that are conducive to sustained smoking abstinence, which could ultimately contribute to lasting public health benefits.

## Data Availability

The data supporting this research are available from the authors on reasonable request.
